# *‘I Believe What I’m Saying More Than the Test’*: The Complicated Place of Rapid, Point-of-Care Tests in Veterinary Diagnostic Practice

**DOI:** 10.3390/antibiotics12050804

**Published:** 2023-04-24

**Authors:** Alison M. Bard, Stephen Hinchliffe, Kin Wing Chan, Henry Buller, Kristen K. Reyher

**Affiliations:** 1 Bristol Veterinary School, University of Bristol, Bristol BS40 5DU, UK; kristen.reyher@bristol.ac.uk; 2 Geography Department, College of Life and Environmental Sciences, University of Exeter, Exeter EX4 4RJ, UK

**Keywords:** veterinary medicine, diagnostic testing, antimicrobial use, antimicrobial resistance, livestock agriculture, qualitative research

## Abstract

Antimicrobial resistance (AMR) is a global health and development threat, with calls for the optimisation of antimicrobial use (AMU) in the treatment of both humans and animals prevalent across national and international policy. Rapid, low-cost and readily available diagnostics that specifically identify pathogens and their antimicrobial susceptibility profiles have been identified as essential parts of this optimisation process, yet questions over the assumed utility of novel rapid technology as a cornerstone of tackling agricultural AMU still exist. To understand whether this technology may support the optimisation of AMU in the treatment of animal disease, this study qualitatively examines the discourse between veterinarians, laboratory representatives, veterinary researchers and (cattle) farmers within three participatory events concerning diagnostic testing on UK farms, to offer a critical examination of the interaction between veterinary diagnostic practice and agricultural AMU. Veterinarian-led discussion suggested that veterinary rationales for engaging with diagnostic testing are nuanced and complex, where veterinarians (i) were driven by both medical and non-medical motivators; (ii) had a complex professional identity influencing diagnostic-test engagement; and (iii) balanced a multitude of situated contextual factors that informed “gut feelings” on test choice and interpretation. In consequence, it is suggested that data-driven diagnostic technologies may be more palatable for veterinarians to promote to their farm clients in the pursuit of better and more sustainable AMU, whilst also being in synergy with the emerging preventative role of the farm veterinarian.

## 1. Introduction

**Figure 1:**
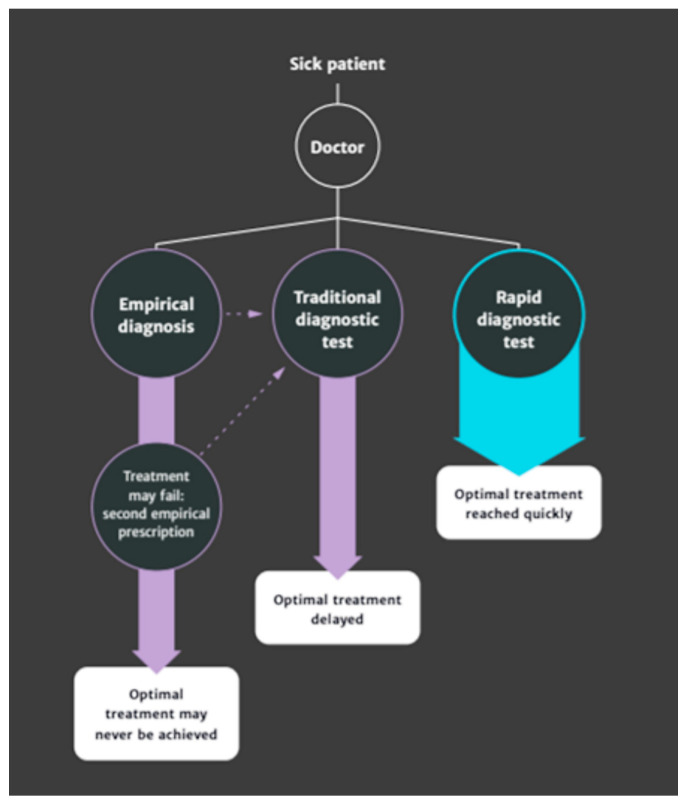
O’Neill’s [2] proposed pathway for how new rapid diagnostic testing would optimise treatment.

The World Health Organisation describes antimicrobial resistance (AMR) as a global health and development threat that requires urgent action [1]. The growth and transmission of AMR is complex, multifactorial and not fully understood; however, antimicrobial use (AMU) is widely recognised as a major driver of AMR [2]. In the pursuit of tackling this global challenge, there is broad consensus that global action must include strategic efforts to optimise the use of antimicrobial agents in the health treatment of both humans and animals [1]. Rapid, low cost and readily available diagnostics have been identified as “an essential part of the solution” in these efforts, given their capacity to inform healthcare providers of the appropriateness of AMU in patient treatment and in driving antimicrobial conservation through optimum treatment choices [2]: Figure 1.

AMU in livestock agriculture is significant given its widespread role in the treatment of infectious disease and, in some countries, in growth promotion [3]. Reducing the use of antimicrobials in animal production and achieving more sustainable methods for ensuring animal health have been widely adopted as policy goals in many countries [4] as a contribution to reducing AMR in both human and animal populations [5]. In the UK, recent efforts in policy initiatives and information campaigns have led to a 55% reduction in overall antimicrobial use in food-producing animals since 2014 [6], indicating significant progress is already being made. However, where antimicrobial prescribing on the basis of clinical observations (rather than microbiological testing) remains the norm, the use of rapid, point-of-care diagnostic testing (RPCT) in identifying biological infection and pursuing treatment of on-farm disease may offer, it has been claimed [2], the possibility of a more targeted and selective use of antimicrobials by “revolutionising farmer and veterinary decision making” [7]. At the heart of this assertion is the idea that RPCT may modify unnecessary AMU on farm by identifying “whether an antibiotic is needed, and which one” [2] in the treatment of animal disease. For example, Malcata et al. [8] assert that the ability to differentiate mild to moderate bovine mastitis caused by Gram-positive bacteria is central to targeted treatment of this condition, with point-of-care testing able to make this distinction. Similarly, Coyne et al. [9] specify that rapid, pen-side tests—able to identify pathogens and their susceptibility profiles—require research, given their potential to ensure antimicrobial use is appropriate within veterinary prescribing practices in the pig industry.

Whilst intuitively logical, this assertion is challenged by recent data on the use of diagnostic testing in UK farm veterinary practice. At present, for many commonly encountered farm animal diseases or infections, specific diagnostic tests are not considered necessary, nor are they regularly used, even when the subsequent treatment involves antibiotics [10]. Moreover, though increasingly available, RPCT do not appear to be central to current veterinary diagnostic practice; there is considerable ambivalence over adopting these technologies, and those that are available are not frequently employed [10]. Indeed, diagnostic practice is viewed as a nebulous, complex process for which rapid tests might constitute only a part [11], posing questions over the assumed utility of novel RPCT technology as a cornerstone of tackling agricultural AMU.

The literature in human medicine suggests that the use and engagement with diagnostic testing is a complex mixture of interlinked biomedical and psychosocial factors, where guidelines need to consider both aspects to alter the usage of diagnostic testing [12]. At present, there has been little qualitative exploration of the circumstances in which veterinarians pursue, engage with and interpret diagnostic tests, an insight that is nonetheless critical to establish both the biomedical and psychosocial factors embroiled in clinical decision making. Without such understanding, recent claims that RPCT adoption offers a means to revolutionise antibiotic use on farm [2] may be overly presumptive and lead to pursuit of technologies that are ill suited to this unique diagnostic context.

This study aims to bridge the knowledge gap around the intricacies of diagnostic-test use within veterinary diagnostic practice through the qualitative examination of discourse between veterinarians, laboratory representatives, veterinary researchers and (cattle) farmers concerning diagnostic testing on UK farms.

The purpose of the paper is to explore (i) under what circumstances veterinarians currently choose to pursue diagnostic testing in veterinary medicine; (ii) what inspires veterinary engagement with diagnostic testing; and (iii) what factors influence veterinary interpretation of these tests. In placing these engagements within the context of specific examples of practice, this paper offers a critical examination of veterinary diagnostic practice and test use on farm and considers whether RPCT may offer the benefits espoused by O’Neill [2] in modifying “unnecessary” AMU in the treatment of animal disease.

## 2. Results

The detailed analysis of the three distinct participatory experiences with veterinarians highlighted three broad diagnostic rationales to explore in greater detail. The first we have termed medical and non-medical motivations. The second is embedded in a rationale of professional identity, and the third is what we have called having value that is fluid and context bound. In the following section, we shall consider these in closer detail.

### 2.1. Theme 1: Medical and Non-Medical Motivations

Whilst discussions between veterinarians in each of the three individual research groups were generally good-humoured and agreeable, there was considerable variability and occasionally discord placed on the purpose of diagnostic texting. Diagnostic testing is well recognised for its implicit medical benefits in aiding diagnosis and treatment decision making (offering clinical direction). However, the discussions held in each participatory event and subsequent analysis suggested that diagnostic testing could be equally useful and appreciated for both identifiable medical and non-medical purposes. In the course of this particular analysis of Theme 1 (Medical and non-medical motivations), we were able to specifically identify how diagnostic testing is seen by veterinarian clinicians as addressing six different functions: direction, emotion, validation, change, prediction and principles.

#### 2.1.1. Direction

A key function of diagnostic testing suggested in these discussions was a means to provide clinical direction, or to identify a causative agent or microbe, in the pursuit of diagnosis:

*“I feel that these tests can be useful for me to reach a diagnosis as well… it’s to get an idea myself… which direction to look for and, I have to admit, I can’t always distinguish clinically which direction it’s going.”*

For this veterinarian, integrating the result of a diagnostic test could be a way to orient themselves in the differential diagnosis process, where the observation of an animal or herd was not always felt to provide them with sufficient information to differentiate between causative conditions that shared similar signs or symptoms. This closely aligns with the medical rationale underpinning O’Neill’s [2] recommendation that a diagnostic test can help a clinician reach the correct diagnosis and, in consequence, infer an appropriate treatment pathway.

#### 2.1.2. Emotion

Using diagnostic testing as a means of confirming diagnosis could also have emotional significance for veterinarians, with discussions suggesting that a veterinarian may “need to have that confidence boost” that a test result can convey as a test “agreeing with you” could create surety regarding the empirical diagnosis. It is possible that this externalised validation, providing evidence that veterinarians could rely on their diagnosis, was needed to allay fear (of making the wrong call or giving the wrong treatment); as one veterinarian indicated ” If you have on-farm culture to back you up then you might be braver”. A confirmatory diagnostic test could also offer a feeling of security to veterinarians, where a result ”could be useful for covering your back”, suggesting a role in avoiding anticipated negative consequences from diagnosis-led decision making on farm.

#### 2.1.3. Validation

A confirmatory diagnostic test could also be valued as a means of highlighting to farm clients that the veterinarian’s empirical diagnosis was correct:

*“It depends whether you are working with a farmer who does not believe what you are saying, so you need to confirm that you are correct in diagnosis.”*

Such a test could also drive a farmer to actively discuss a topic by making an empirical diagnosis a visible reality:

*“The only reason I’d do that test is not to find out what’s wrong with that cow or to persuade myself it’s ketotic, it’s to get a ketotic result. I’d go to a farm and say, “Look at this, you’ve got to engage with me to discuss this problem”.”*

Both these veterinarians appeared to use diagnostic test results as a means of validating their veterinary expertise in the farm client’s eyes, with this evidence perhaps less likely to be questioned or argued with than their own empirical interpretations. In doing so, they felt this would be more likely to foster farmer engagement with the diagnosis and recommendations at hand.

#### 2.1.4. Change

Other veterinarians suggested that a diagnostic test result was useful when, and only when, the resulting information would initiate a change in behaviour or action, being “only worth doing if it’s going to change what you’re going to do” or “only useful if an action comes out”. In this instance, the formation of a diagnosis was not a sufficient motivation to test without the assurance of a resulting action or effect. For example, in considering mastitis, veterinarians discussed the lack of value in using a diagnostic test to establish a diagnosis-relevant treatment protocol if they felt they would be pressured to define and prescribe a treatment protocol before the test result was returned.

#### 2.1.5. Prediction

Diagnostic tests were also reported to be used for prognostic purposes, where a diagnosis may already be clear but is enriched by the additional detail a diagnostic test result can provide when compared against previous test–disease–outcome relationships:

*“I’m really interested now in using the diagnostic results for prognostic purposes. I want to try and understand at what level of anaemia you’re likely to get a response or not… To me, there is a value potentially… I will try and get a sense of whether [client’s animals] can make it or not.”*

A test might also be used to assess the severity of a known diagnosis, to predict treatment outcomes:

*“But yeah, I do BHB’s [beta-hydroxybutyrate test] when I suspect a ketotic cow. I guess, like you say, it’s off food, it’s freshly calved, so it’s going to be ketotic, but if I know how badly ketotic it is, it might change how I treat it?”*

Going a little further by interpreting diagnostic test data to predict the future health of the animal or establish nuance in a diagnosis was seen as personally rewarding by veterinarians, whilst also offering additional assurances to farm clients and more specific/accurate treatment to animal patients.

#### 2.1.6. Principles

Veterinarians reported that it was not always the pursuit of definitive diagnosis that was relevant to enacting a diagnostic test, rather, it was the confirmation of a diagnosis already made. This was due to a principle or particular logic of practice governing their behaviour, such as “I believe testing should be carried out to confirm a diagnosis not to make a diagnosis”. For this veterinarian, it was their own expertise and empirical diagnosis that was at the heart of veterinary decision making, where they suggested relying on a diagnostic test result to make a diagnosis could cause unnecessary delays that could impact on animal welfare: “When you go and see a downer cow … shoot it. Don’t take a blood sample and three days later get a list of results this long and tell the farmer it’s dying of liver failure so we’ll shoot it”.

### 2.2. Theme 2: Enmeshed with Professional Identity

Veterinary discussion also suggested that the purposeful pursuit of diagnostic testing and diagnostics represented something essential about being a veterinarian, with testing being connected to the responsibility and privilege of diagnosis. Diagnosis is a key deontological territory for veterinarians and the greater accessibility and democratisation of rapid and on-farm point of care diagnostic tools was interpreted as a potential weakening of on-farm veterinary roles.

*“There’s a fear with [on-farm culture plate system] on the farms I work with–they’re under a lot of pressure to become a technician, become involved in making the decisions … I think within a few weeks I’d be cut out of the loop and he would be the diagnoser and the treatment instigator and making bad decisions… So us vets are always at risk… the more we kind of downgrade what we do, the more we’re side-lined. I think that’s a real big problem–I know which farm I’d like to use it on but I don’t think I’m going to use it on it ‘cause he’s already doing mass formal protocols for reproduction. So all the skill on my [reproductive] scannings go and now this would just be another thing.”*

Indeed, in discussions with farmers in Event 3 regarding reviewing on-farm culture plates, this tension appeared to arise in differences in how much the veterinary facilitators wished their farmers to take on the role of “diagnoser” when reviewing plated samples:

(Vet) *“Yeah so anyone want to comment on that one [culture plate]?”*

(Farmer) *“Klebsiella?”*

(Vet) *“So I think this is where me and [other vet] differ. I mean, I don’t encourage you to interpret so much…”*

Here, the veterinarian was explicit in drawing a line under where she felt the farmer’s responsibility ended in the process of diagnosis through RPCT; she was comfortable with broad interpretation (Gram-negative or Gram-positive bacteria) to inform treatment regimes, but not comfortable with farmers providing the finer details that pervaded the veterinary identity as “diagnoser”. In contrast, her veterinary colleague was perceived to be more comfortable encouraging clients to interpret the RPCT samples in this more complex way, suggesting variation in perceived identity vulnerability through sharing “diagnoser” behaviours with farm clients.

Veterinarians reported that “bundling” on-farm testing and veterinary advisory support in a package (e.g., pay per month for tests and veterinary time) could mediate their perceived vulnerability in letting go of the “diagnoser” role through RPCT—"so the only way farmers get [the tests] is if they are involving you in the loop”—in addition to targeting those farmers with whom they have the most trusting relationships for on-farm RPCT schemes, where they would know that a client would effectively follow their veterinary recommendations:

*“There needs to be a trust circle. You need to have a protocol that says if it’s there, you give it that and trust the farmer to do it. If you don’t trust them there’s no point doing it.”*

Indeed, the veterinarians running Event 3 emphasised the niche utility of RPCT for specific clientele, depending on their personalities, time constraints and ability. The construction of this on-farm culture group reflected this assertion, with those farmers attending having received a specific invite as individuals chosen to work with on-farm culture in collaboration with the veterinarians. Veterinarian–farmer interactions throughout appeared to reflect familiarity rather than formality in broaching topics related to testing and treatment perspectives, suggesting positive relational ties between veterinarians and farm clients involved (e.g., use of humour and laughter [13]; sharing emotional responses [14]—this environment may be as crucial in disseminating RPCT and delegating part of the “diagnoser” identity.

This link to the purposeful “diagnoser” veterinary identity that may influence the sharing of RPCT was also reflected in veterinary attitudes towards testing that involved little or no self-direction on behalf of the veterinarian. In these instances, veterinarians appeared to mentally detach themselves from the testing process. For example, in participatory Event 2, when one veterinarian voiced the idea of including (government-mandated) bovine tuberculosis testing as a part of clinician diagnostic-test data, this was met with raucous laughter from the entire group, as if the idea of a veterinarian claiming personal ownership of this diagnostic practice was entirely comedic. Additionally, when discussing test results from (certification-mandated) Johne’s disease tests, one veterinarian suggested this somehow did not feel a legitimate representation of their diagnostic practice as it did not relate to their own decision making:

*“I was saying actually a lot of the Johne’s tests we get back, it really isn’t fair. It’s not even a question of being fair, but it massively skews the data if every quarter I get [retailer] Johne’s result back, but it’s not my decision to do that test. It’s [retailer]’s decision to do that test.”*

Here, the “testing data” attributed to this veterinarian within the practice system did not appear “fair”, in being incongruent with their self-directed decision making.

It is important to note that this purposeful pursuit of veterinary agenda and diagnosis could encompass both monitoring and diagnostic forms of testing; for example, veterinarians attending Event 2 highlighted colleagues with a particular interest in infectious disease who were particularly enamoured with monitoring diagnostic processes. Rather than being linked specifically to the type of test and desired outcome, tension or disengagement in all groups appeared to arise over the decision to test being externally sanctioned or outside of their control.

### 2.3. Theme 3: Value Is Fluid and Context-Bound

**Figure 2:**
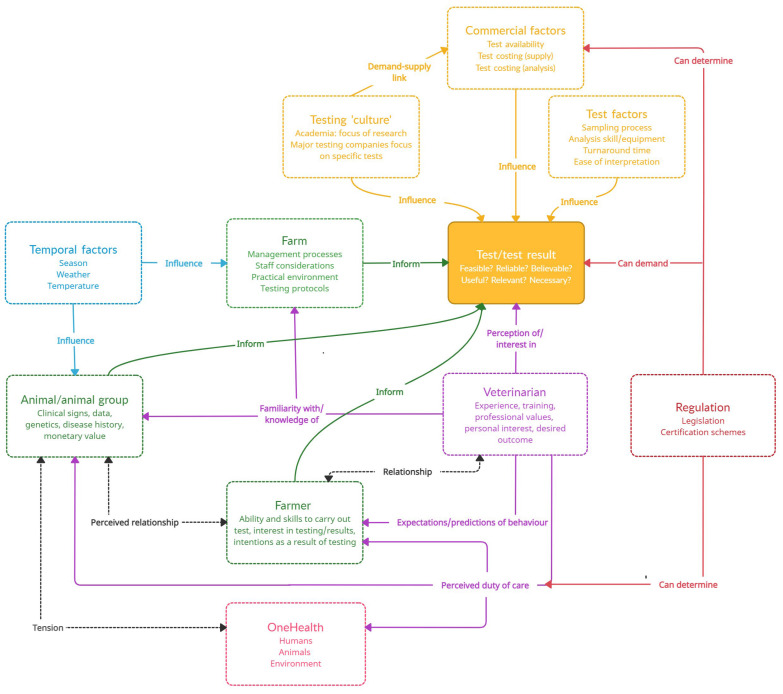
Intertwined circumstantial factors that act to calibrate veterinary diagnostic practice. * Green: farm factors, yellow: test factors, purple: veterinarian factors, blue: temporal factors, red: regulatory factors, pink: One Health factors, black: relational factors.

Diverse factors calibrated veterinary diagnostic practice (Figure 2). These extended beyond the traditional diagnostic pillars of farm and animal health presentation/history to those relating to the test itself: seasonal considerations, regulatory influences and the positioning of the veterinarian themselves (i.e., relating to their internal drivers, contextual perceptions and perceived relational expectations; Figure 2). There did not seem to be any overarching “diagnostic hierarchy” with regard to specific factors always being more or less important in selecting diagnostic tests. Instead, veterinary diagnostic practice was situated within the complex interplay between these factors, meaning that the value of a test was often circumstantial and context-bound rather than contingent on the test itself. Indeed, there was considerable ambivalence at a group level (Event 2) when veterinarians were polled on the absolute value of any individual test or test result (e.g., red blood cell count, coccidiosis count, creatinine) given the complexities of these circumstantial considerations; instead, the veterinarian presenting on diagnostic-test use was left summarising the attempts to find any consensus on value as “it’s so complicated.”

Discussions instead suggested that the wide variety of contextual considerations intertwined to create a gestalt diagnostic impression, an overall sense of gut feeling or intuitive response about the animal health challenge and appropriate assessment, created from the combination and balancing of these differing contextual factors (e.g., “My gut feeling would be that PCR [polymerase chain reaction] would give you a better answer for that [high cell count cattle mastitis]”. It was against this gestalt impression that the benefit of instigating a testing process was weighed or the reliability and believability of a test result was assessed, making the potential value of any test fluid depending on this context and the veterinary interpretation thereof. Test data was therefore viewed as one part of a “big picture” of what might be going on within any farm, not (necessarily) a determinant where a result without interpretation by a veterinarian could even be considered “relatively meaningless.”

Indeed, veterinarians involved in Event 3 reflected the value they placed on this flexible integration of tests and test outputs in a gestalt diagnostic impression when training farmers in how to assess and respond to mastitis on-farm culture. Here, veterinarians encouraged farm clients to cultivate this gut feeling when making their own assessments of testing and treatment processes, in the pursuit of effective decision making:

*“So it might be that you’ve got to use your gut and your common sense that actually if it really isn’t responding in a way that most of them do, then you start to question … We’ll re-sample and perhaps double check with us but probably, yeah, pragmatically you just say, “Right I’m just going to give her a tube and do her a course so that we don’t get what we don’t want”.”*

In some circumstances, this gestalt impression could even act to outweigh a test result that was misaligned with the overall gut feeling experienced by the veterinarian: “Because I believe what I’m saying more than the test.”

Nevertheless, veterinarian checks and balances on how individuals integrated testing and test outcomes within decision making were complex, despite accepting this fluidity in test adoption and interpretation. Veterinarians in Event 2 discussed the issues concerning truly knowing the on-farm outcomes of testing and treatment decisions, given inconsistent within-practice data recording. For focused “diagnostic” testing—most often a temporarily distinct assessment of disease in response to an immediate health challenge—both animal cure or culling/death may not necessitate a data record with the practice if there was no direct formal veterinary consultation on these outcomes (which may, depending on the case, be discussed informally with the farmer at a next visit or not at all). Additionally, the exact treatment decision made by the vet in response to a test may not be apparent from the records on the test itself. As the colleague who had collated empirical data on all diagnostic testing carried out within the practice over the six-month period reported:

*“Some of that [the relationship between test use and treatment decision making] I can’t really get out of the data because all I get is a result, I don’t get what the vet then did with that result… I don’t know what happened there afterwards.”*

For “monitoring” testing—most often a repeated, temporally spaced routine evaluation to inform an ongoing picture of animal health—checks and balances may arguably be embedded in the testing procedures themselves. Gathering test data of this kind, when used to evidence the occurrence of a specific disease challenge and assess trends or cases over time, could, by design, encourage personal veterinarian reflection on decision making by pinpointing changes following any advised treatment or management pathway. However, for this sort of critical assessment, it is data continuity that is critical, something reported as often “*frustratingly*” absent within practice records across testing types:

*“* *So one of the things that’s been really frustrating for me, is often, not so much if we submit to an external lab because we’ll tend to write a number on, but if we submit to our lab, the amount of tests that just have “sick calf”… something that’s tested at four months in January and at eight months old in April, I’ve no idea if it’s the same animal or a different animal because there’s no continuity.* *”*

Whilst there was acceptance of this issue in Event 2, this was felt by a responding veterinarian to simply be indicative of clinical services being the priority within veterinary practice, combined with a lack of guaranteed veterinarian interest in seeking out these data:

Responding vet: “I take your point entirely… but our primary reason for being is to provide clinical services as a veterinary practice to our clients and not to provide research data.”

Initial vet: “But if we have better case continuity, then surely that in itself would provide a better clinical service to our clients…”

Responding vet: “And I agree…Who out of us, if we went to see an individual sick calf at eight months old, would go back and look through the notes and see whether they had been tested?”

Better integrated technology systems were suggested as a solution to this issue, that would allow veterinarians to check on farm “what someone had done the day before, the week before, or whatever, with that same animal”, facilitating better transparency of the context-bound decision making carried out by veterinarians and thus establishing continuity and accountability within the diagnostic data.

## 3. Discussion

The exploration of veterinary perspectives in diagnostic-test usage suggested that the rationales behind adopting diagnostic tests could extend from medical to non-medical motivations (direction, emotion, validation, change, prediction and principles). This pursuit of diagnostic testing for non-medical purposes is also reflected in human medical services, where UK general practitioner (GP) narratives suggest comparable motivators to engage in diagnostic testing beyond the instrumental: as “defensive medicine” to avoid litigation; as reassurance for a nervous patient; as a “tactical” procedure to persuade of a diagnosis; as a habit or personal routine established in medical education; as a response to needing to offer something useful to a patient when having “empty hands” (lack of diagnostic or therapeutic plan); or as a “magic ritual” where patients place high trust in diagnostic testing results [15]. In a study of blood testing by UK GPs, Watson et al. [12] suggested non-medical rationales were underscored by two overarching motivators: managing uncertainty (e.g., litigation, the unknown) and providing a “gift” to patients (e.g., reassurance, insight, showing acknowledgement of concerns).

In re-emphasising the link between better diagnostic-test use and improved antimicrobial decision making as a medical and linear progression—as can be seen in O’Neill [2] regarding “whether an antibiotic is needed, and which one”—it may be tempting to view non-medical motives for diagnostic testing as in some way “inappropriate” or “unnecessary” as they are divorced from this instrumentally oriented framing. This is, however, not borne out or supported by examples and analysis from practice. As Van der Weijden et al. [15] concluded with regard to GPs, “non-medical motives may be just as rational and legitimate in the overall context of a particular patient’s care as the medical decision-making process”. That is, in the promotion of effective patient care, both biomedical and psychosocial elements are of value [12]. In the current study too, veterinarians echoed this sentiment, recognising that diagnostics provided them with individual psychosocial benefits (e.g., bravery, confidence) in addition to benefits to the client; specifically, one participant in Event 1 suggested that, in providing diagnosis and treatment, “you are treating the farmer as well as treating the animal.”

We maintain that these non-medical motivators of diagnostic-test use may not only be both “appropriate” and “necessary” when considered in psychosocial terms but may also have benefits with regard to veterinary communication with farmers regarding the utility of RPCT in the unique situated reality of prescribing and antibiotic use on UK farms. In the UK context, veterinary clients have the privilege of storing and administering medicines prescribed by their veterinarians to their animals [16]. This responsibility means that farmers are often faced with the decision to treat (or not); something which is described as involving “bravery” (Event 1). This is of little surprise given that antibiotic use has been documented to be conceived by farmers to be “*cheaper than a dead cow*” [16]. Veterinarians’ honest framing of RPCT use as a means of emotional support to counteract the “*overwhelming*” (as described by a farmer during Event 3) feeling of holding back antibiotic treatment may do more for farmer engagement with RPCT and sustainable usage of antibiotics than if veterinarians’ narratives focus on medical rationales alone.

Veterinarians’ narratives on test usage suggested that many factors competed and/or contributed to testing engagement (Figure 2), with veterinary diagnostic practice situated within the complex interplay between these factors. There did not seem to be any overarching diagnostic hierarchy with regard to specific factors always being more or less important in selecting diagnostic tests, meaning the value of a test was often circumstantial and context-bound rather than contingent on the test itself. These complex and intertwined factors—with no overarching hierarchy of value—are similarly echoed in the medical literature concerning the construction of medical diagnosis. Berg [17] argues that diagnoses are transformative processes between medical data, the image of the patient and social factors: *“In these fundamentally reciprocal processes, no fixed hierarchy exists: examination results do not necessarily count more than historical [results], data do not, in principle, overrule interfering social factors, etc.”*

A recent questionnaire examination of motivators for diagnostic-test use by UK veterinary surgeons (*n* = 153), [10] may, on initial examination, appear to contradict the lack of diagnostic hierarchy suggested in these participatory event data. Veterinarians consistently valued specific circumstantial factors when rating their influence on diagnostic practice: for example, regulatory factors were predominantly rated as always or often influential, whilst the sentimental value of an animal was predominantly rated as rarely or never influential. However, even within the categories of this survey, nontrivial ambivalence was witnessed; whilst 59% of veterinarians stated regulatory factors always influenced a diagnostic testing decision, 22% stated this was rarely or never so [10]. In this circumstance, either >20% veterinarians choose to either act unlawfully by rarely or never considering regulation, or—arguably, more likely—veterinarians responding were considering differing testing environments and animal health circumstances, which for some veterinarians included regulation and for others did not. It would appear that the contextual influences scaffolding any diagnostic practice and particular testing decisions are often not easy to compare nor attribute value to between veterinary diagnostic experiences, and whilst Chan et al.’s [10] data indicate variability, they are unlikely to indicate hierarchy.

Discerning absolute diagnostic priority or hierarchy—such as within clinical guidelines or standard operating procedures for test use—within the intertwined contextual factors implicit in everyday veterinary diagnostic practice is therefore challenging. The combination and balancing of these contextual factors is likely specific to individual clinical cases, even when considering specific disease trajectories. However, the nuanced and variable inclusion of contributing factors, weighed situationally to inform test choice and interpretation, do not make the diagnostic endeavour reported any less scientific merely because it cannot be easily constrained into a protocol, decision tree or guideline. Indeed, as highlighted by Berg [18] on medical diagnosis, “An explicit statement (e.g., a diagnosis) can be the end result of a period of work, but that does not mean that the process of producing the diagnosis itself can adequately be represented as a series of explicit statements.”

Moreover, in attempting to define such hierarchies linked to treatment outcomes, Berg [18] argues that the resulting protocols or guidelines—sets of instructions telling medical personnel to do “A” in “B” situation—will actually serve to create problems, as they naturally focus on data and interventions that are easily represented (a test, procedure or threshold) and ignore those that are inherently more difficult (a farmer–animal relationship, a personal history) as evidenced in the authors’ attempt with mastitis following participatory Event 1 (Supplementary Materials Figure S1). This process of designing decision support tools can, in itself, be problematic, where an artificial hierarchy is created simply by consequence of the more “*formalisable*” matters of diagnostic criteria being made explicit, leading to the devaluing and de-emphasis on that which cannot be made so; a set up “which can lead to the unacknowledged loss of valuable information and interventions” [18].

The absolute and separable diagnostic behaviours and their associated treatment outcomes posited by O’Neill (Figure 1; [2]) and used to illustrate the relative advantage of RPCT in antimicrobial stewardship, therefore, not only become somewhat tenuous as a solution to identifying “whether or not an antibiotic is actually needed, and which one” in the diagnosis and treatment of many farm-animal-health challenges but are perhaps damaging in their simplicity. It is perhaps for this reason that veterinarians do not currently view RPCT as a panacea for the unnecessary use of antibiotics in veterinary treatment, despite recommending disease foci embroiled with antibiotic use as areas where RPCT are needed (e.g., mastitis, [10]). Indeed, in Chan et al.’s [10] work, RPCT were reported by veterinarians “as one more bit of information for the interpretation of the clinical case, when used by the veterinarian, in the decision making for the use of antibiotics and for which antibiotics to use”. Acknowledging RPCT as one component within complex decision making—rather than an authority on antibiotic use in and of itself—would perhaps lead to greater acceptance and engagement of new technologies on farms.

Further complicating this picture appears to be the sometimes intuitive, gut-led responses that veterinarians report, where a gestalt diagnostic impression drives case assessment, test use and interpretation. This gut response appears to be one way to combine and balance the myriad contextual complexities implicit in any veterinary decision making surrounding an animal-health challenge (Figure 2), as without a clear hierarchy in the diagnosis and testing processes, there is no simple way to rationally weigh up the importance of each factor against another when determining appropriate diagnostic practice. This reliance on gut feeling is mirrored in human medical contexts, where an analysis of GP reports suggests these gut feelings either direct individual diagnostic efforts via a sense of “alarm” (an uneasy feeling of “there’s something wrong here”) or a sense of reassurance (a secure feeling of “everything fits in”), casting either doubt or confidence on testing outcomes, respectively [19]. This propensity to gut feelings is explained as the balance between intuitive and analytical reasoning—when there is dissonance, an internal gut feeling or belief can arise that something is wrong despite a lack of clinical markers [20]. As one veterinarian in the present study reported “because I believe what I’m saying more than the test.” The internal diagnostic compass can outweigh an external knowledge source.

Veterinarians in this sample indicated that current data-recording processes may make examining and reflecting on the accuracy of these gestalt diagnostic impressions difficult, given the lack of continuity and thus accountability within the testing data. Within empirical research, there is a dearth of studies examining the accuracy of gut feelings on clinical outcomes, with those in the literature displaying directly conflicting results. For example, Van den Bruel et al. [21] found that the gut feelings of GPs identifying something wrong in children—even when unexplained by clinical assessment—had high specificity and a high positive-likelihood ratio for serious infectious illness. Conversely, Turnbull et al. [22] found that clinician gut feeling was not a good predictor of a child’s illness getting worse (that is, either resulting in a return to the GP or being admitted to hospital) with regard to respiratory tract infections (although gut feelings were predictive of both antibiotic prescription and referral). Gut-feeling accuracy does not seem to be well understood in the medical diagnostic literature and—to these authors’ knowledge—is not examined at all within veterinary diagnostic literature, despite reference to gut feeling in veterinary textbooks [23] and journal publications [24] as a source of clinical insight/information. Further research is needed to understand how gut feeling may act to influence diagnostic practice in veterinary work and its associated impacts on diagnostic testing and antibiotic prescription.

The place of RPCT in reducing unnecessary antibiotic use is further complicated by the potential linkage of diagnostic testing and veterinary identity, with the participatory event data suggesting diagnostic testing may hold symbolic meaning for veterinarians. The literature exploring the formation and consequences of veterinary identity (i.e., values and priorities that are meaningful and which guide and inform professional behaviour) suggests understanding this identity is important for veterinary mental health and coping [25], where identity self-understanding (I know what is important to me) and identity-behaviour alignment (I can remain true to myself in my actions and decisions) contribute to positive psychological health [26,27]. Research in veterinary identity is in its infancy; however, Armitage-Chan et al. [28] suggest that veterinarians foster two differing characterisations of identity from graduation, either (i) an academic and “diagnosis-focused” identity, where individuals place greatest value on successful patient diagnosis and treatment with a curative mindset or (ii) a “challenge-focused” identity, where individuals prioritise technical competence and decision making in its contextual complexity (clients, colleagues and business).

For those veterinarians who more readily align with a curative, “diagnosis-focused” identity and role, the contextual complexities of lived veterinary practice can be seen as frustrating obstructions to the realisation of these identity goals (animal healing), even stimulating feelings of anxiety, instability and lack of control due to “identity dissonance” [29]. It is possible that these negative feelings may be acute in considering the sharing of RPCT with clients, as it is the very tools of diagnosis—intimately connected to the Aesculapian authority to heal animals embodied by this curative identity—that are being shared with a non-veterinarian, thus obstructing the veterinarian’s own curative role. It is no surprise that these tools, then, were discussed by the veterinarians as only being shared with specific clients with whom veterinarians experienced trust and expected competence, with discussion on sharing rapid diagnostics evoking words such as “risk”.

This “diagnosis focus” component of veterinary identity may therefore complicate efforts to disseminate RPCT in farm animal practice, given the potential symbolic meaning of tools identifying a specific causative agent or microbe within veterinary value systems. Indeed, this may contribute to veterinarian ambivalence about the place of RPCT itself [10]. Successful veterinary engagement with the distribution of these technologies to farm clients may therefore require interventions aiming to foster complimentary veterinary values as much as addressing practical considerations surrounding dissemination, such as test and market conditions. For example, in the Arwain Vet Cymru complex antimicrobial stewardship intervention [30], where participants were encouraged to become active stewards of antibiotics as “Veterinary Prescribing Champions”, some participants focused on providing greater opportunities for farmers to use RPCT [31] as a means of fulfilling this professional identity. By inviting veterinarians to actively characterise antimicrobial stewardship as part of their identity self-understanding, surrendering the tools of diagnosis to a broader pool of farm clients was possible whilst remaining true to their professional veterinary identities.

Alternatively, data-driven diagnostic technologies that can detect health issues at an early stage and help ensure optimal environmental conditions [32] may be more palatable for veterinarians to promote to their farm clients in the pursuit of better and more sustainable use of veterinary antibiotics. Firstly, these “smart” or “precision” technologies are not so intimately connected to the act of veterinary diagnosis (i.e., do not identify a specific causative agent or microbe) but instead seek to enhance and sharpen farmers’ existing wealth of skills and knowledge in observing animals’ signs of disease. In alerting farmers to changes in animal or environmental signs, these technologies therefore surrender the action of *prognosis* to farmers (What is likely to happen next given these data? Is there an impending health challenge?) rather than diagnosis (What specific disease is this?), ensuring the implications of the technology are less identity threatening for “diagnosis-focused” veterinarians. Secondly, farmer engagement with these technologies would act to strengthen the veterinarians’ own diagnostic practice, by providing better data on situated farm-level considerations that scaffold veterinary-decision making. Third, these data-driven diagnostics technologies appear to match more closely with the newly emerging preventative role of the farm veterinarian [33] than RPCT, by providing veterinarians with complex data that enable them to anticipate potential health challenges and advise management adjustments accordingly (thus avoiding future antibiotic use), rather than *react* to disease incidence on farm.

A focus on data-driven diagnostic technologies as the new veterinary diagnostic frontier for reducing unnecessary antibiotic use on farm may, therefore, be more intuitive than the RPCT heralded for this purpose by O’Neill [2]. However, these technologies may have implications for both animal welfare and human–animal relationships [32] and there is a dearth of research examining the application and perception of these technologies by veterinarians and farmers within the differing UK livestock sectors, each with unique considerations. Further research is needed to explore the relationship of these data-driven technologies with antibiotic use in animal production.

## 4. Materials and Methods

To explore the perceptions, experiences and understandings that underpin UK farm veterinarian diagnostic practice and test use, this methodology details the recruitment and qualitative analysis of three participatory events (i.e., professional gatherings where veterinary participants shape the agenda before and during the event) typical to UK veterinary practices. Each participatory event was selected to offer a differing perspective on veterinary diagnostic practice, with all participatory events occurring between March and June 2018.

### 4.1. Sample Recruitment

Given the complex and often context-dependent nature of diagnostic-test use, the opportunity to observe and analyse discussion *between* veterinarian colleagues on this topic—with their ability to challenge, question, empathise and probe one another using their own insights and from their own experience as veterinary practitioners—was felt to offer key insights to diagnostic-test use. Furthermore, the observation and analysis of veterinary colleagues with existing professional and/or personal ties, where communication behaviours that typify workplace interactions (such as sarcasm and humour, [13]) would naturally occur, were identified as having the greatest potential to foster honest, transparent discussions on diagnostic-test use and treatment decision making. For this reason, the research team aimed to recruit to the project a selection of diagnostic-testing-oriented participatory events already established within existing UK veterinary teams, given the rich interpersonal dialogue resulting from these particular group dynamics that may not be easily replicated using other research approaches (e.g., one-to-one interviews, focus groups of participants without existing social ties).

Following the discussion of participatory event opportunities within the research team, three types of veterinary practice discussion contexts were identified as offering interactional dialogue that would typify a standard veterinary practice experience, whilst at the same time offering qualitatively differing perspectives on the complexities of diagnostic practice and testing in the farm context (Table 1).

Seeking out opportunities to access and attend one participatory event from each discussion context, the research team approached contacts within the veterinary industry to identify any upcoming generic veterinary group meetings within existing professional networks and businesses. Three participatory events were recruited for the purposes of this study, reflecting these three discussion contexts, with each attended by the research team and recorded using an Olympus DS-3500.

### 4.2. Ethics Statement

The study obtained ethical approval from the University of Exeter Geography Ethics Committee (approval reference number eCLESGeo000069v.3.0) ensuring procedures met ethical guidelines in place for research with human participants. All participants were informed of the purposes and process of the research study and written consent to take part was obtained. Given the ethical concerns about the likelihood of the identification of participants from each meeting—given the limited geographic context and the links of the participating practices to the known research team—specific demographic details of participants are not included in this methodology to ensure the privacy rights of the human subjects are suitably observed.

### 4.3. Participatory Events

Context 1: The first participatory event comprised five cattle veterinarians on a “mastitis expert panel” within a large UK veterinary corporate (i.e., a group of practices owned and operated by a company) along with one laboratory representative from one of the UK’s leading full-service veterinary laboratories, who wished to create a diagnostic guide for veterinarians within their company to use regarding the diagnosis and treatment of bovine mastitis. Facilitated discussion focused sequentially on the exploration of: (i) which mastitis diagnostic tests, in which circumstances, and why; (ii) decisions in response to mastitis diagnostic tests; and (iii) prescribing and treatment responses in mastitis (3.5 h total).

Context 2: The second participatory event consisted of 12 veterinarians who had organised a “clinical club” (informal practice meeting) within a veterinary practice to discuss the research of a colleague who had collated empirical data on all diagnostic testing carried out within the practice over a six-month period (including all rapid, in-house and laboratory testing). The veterinarian responsible for the research wished to use the clinical club as a platform to discuss the validity of the collated/analysed diagnostic test data along with the perceived utility of the common testing processes undertaken within the practice. Hypothetical “case scenarios” were also discussed to evoke individualised diagnostic practice in the farm context (“the down cow”, “suspect ketosis cow”, “scouring calves” and “scouring lambs”) (1.5 h total).

Context 3: The third meeting consisted of two cattle veterinarians, three veterinary diagnostic researchers and 11 cattle farmers, the latter of whom were currently engaged in on-farm bacteriological testing of milk to guide antibiotic treatment-decision making in suspected mastitis (“on-farm culture”). The meeting included four parts, led by the cattle veterinarians as facilitators: (i) an educational overview and group discussion of mastitis occurrence, diagnosis and treatment in dairy cattle; (ii) a veterinarian-led practical examination of dissected bovine udder tissue; (iii) a veterinarian-led practical examination of on-farm culture plates brought by participating farmers; and (iv) a participatory discussion on farmer experiences of adopting on-farm culture in the management of bovine mastitis (3 h total).

### 4.4. Considering Validity

Recruitment in this study created a specific grouping of participatory events; Events 1 and 3 explored diagnostic testing in the dairy context for bovine mastitis, whilst Event 2 oriented around all diagnostic testing carried out by a practice engaged in the diagnostic testing of dairy, beef, sheep, camelids, pigs, deer and birds. The cohort involved in this study were also all participants currently working within the South and Southwest of the United Kingdom. As such, the veterinary perceptions and understanding conveyed in the contextual discussions may have been shaped by these group dynamics and participant experiences.

Non-probability sampling of this kind (as well as qualitative analysis processes) can never claim to deliver a universal, representative picture of veterinary diagnostic practice and testing, yet they offer nuance and understanding with which positivist methodologies are ill-suited to grapple. Given the routine and common nature of the three veterinary discussion contexts identified (Table 1) to drive the selection of these participatory events, this study was designed to provide detailed, rigorous insights of value in considering veterinarians’ diagnostic practice and testing experiences. Common features of each participatory event (i.e., participants having existing personal/professional ties, meeting in an informal setting and having a special interest in the event topic) created a fertile environment for honest and critical, yet socially comfortable explorations of perceptions and ideas, where challenges and queries to ways of thinking that could not have been made in so forthright a fashion by an unknown researcher were made between participants with ease. In this way, the rich interpersonal dialogue offered a unique window on diagnostic practice and testing of relevance in consideration of the adoption of RPCT.

### 4.5. Analysis

Participatory events in the three discussion contexts generated data from the interactions between group participants. It was attention to these interactions that was critical in the analysis process, as typified in focus-group methodologies where examination of complex group dynamics allows the exploration of perceptions, experiences and understandings [34]. Thematic analysis was chosen as a methodological framework for participatory event data as it can be applied flexibly, without a single a priori theoretical assumption about what may be learned from the data. This process was informed by Braun and Clarke’s [35] iterative thematic analysis steps (data familiarity, generating initial codes, searching for themes, reviewing themes, defining and naming themes, producing a report) following what may be most closely termed as a “Reflexive Thematic Analysis” [36].

Unlike “coding reliability” approaches to thematic analysis—exemplified by the work of researchers such as Boyatzis [37] and Joffe [38]—RTA aims to fully embrace the qualitative research values and subjective skills a researcher brings to the thematic analysis process, where “a research team is not a required or even desirable quality …. meaning and knowledge are understood as situated and contextual, and researcher subjectivity is conceptualised as a resource for knowledge production” [36]. That is, analysis is conceptualised as a situated and interactive process, reflecting the data, the positionality of the researcher and the context of the research itself. As such, RTA was completed in full by AMB through open and organic coding, with themes being a final “outcome” of this organic data coding and iterative theme development [36]. Other authors provided input through informal discussion and reflection on this process as it unfolded, given their familiarity with the transcripts and/or research topic.

This analysis process was fundamentally exploratory and subjective, involving active, creative and reflexive researcher engagement [35]. The following broad steps were followed, with repeated re-evaluation of codes, themes and raw data: (i) familiarisation with data (hard copy) examining similarities, patterns and ideas; (ii) development of initial “codes”, i.e., succinct labels identifying what was of interest in the data in relation to the research questions; (iii) codes summarised, shared and discussed with the research team; (iv) transcripts imported into NVivo software for the purposes of re-reading and re-examining foundation codes; (v) using codes as “building blocks”, coding and transcript data examined for common, recurring patterns across the dataset that could be identified and clustered around a central organising concept or idea (theme); (vi) organising themes named and reviewed in relation to the initial codes and the full data set, refining the characteristics of each theme; and (vi) results produced, using the process of writing in itself as an analytic and creative process, stimulating further critical assessment of the thematic structure.

## 5. Conclusions

O’Neill (2016) suggests that the emergence of novel RPCT may be a way to more effectively target whether an antibiotic is needed—and which one should be used—in the provision of veterinary medicine. However, veterinarian-led discussion surrounding diagnostic-test use suggests that veterinary rationales for engaging with diagnostic testing—in addition to the interpretation of resulting test data—are nuanced and complex. Veterinarians (i) are driven by both medical and non-medical motivators (direction, emotion, validation, change, prediction and principles) when engaging in testing processes; (ii) have a complex professional identity influencing diagnostic test engagement; and (iii) balance a multitude of situated contextual factors that inform “gut feelings” on test choice and interpretation. Data-driven diagnostic technologies that are not intimately connected to the act of veterinary diagnosis (such as “precision” or “smart” farming technologies) may be more palatable than RPCT for veterinarians to promote to their farm clients in the pursuit of better and more sustainable use of veterinary antibiotics, whilst also being in synergy with the newly emerging preventative role of the farm veterinarian. Further research is needed to explore the relationship of these data-driven technologies with antibiotic use in animal production.

## Figures and Tables

**Table 1:** Three veterinary practice discussion contexts identified for participatory event recruitment and their potential perspective on diagnostic practice and testing.

Veterinary Practice Discussion Context	Potential Participatory Events	Perspective Offered
**Context 1** Veterinarians’ discussions with colleagues in pursuit of a common goal	Setting up a code of conduct for medicine use, or decision tree to guide diagnosis/treatment of specific disease challenges	To foster collegiality and evoke “collaborative diagnostic logic” *Diagnosis and treatment as discerned by a veterinary team seeking common practice*
**Context 2** Veterinarians’ discussions with colleagues in pursuit of an individualised goal or perspective	Within-practice discussion of diagnostic testing protocols established with farm clients, or veterinary training on diagnostic testing	To evoke “personal diagnostic logic” *Diagnosis and treatment as discerned individually, given unique experiences and farm client interactions*
**Context 3** Veterinarians’ discussions with farm clients	Within-practice training/knowledge exchange with farm clients in: *Medicine use* *Rapid diagnostic-test use* *Specific disease assessment, testing and treatment*	To evoke “shared diagnostic territory” *How diagnosis and treatment knowledge are shared with farm clients, highlighting where opportunities and tensions may arise for sharing of diagnostic technologies*

## Data Availability

Data in this study are not readily available due to ethical restrictions. Given the personal nature of participatory event discussion material, participants consented to material derived being used only for scientific and research purposes/outputs coordinated by the research team. As such, datasets generated are deposited in a UKRI depository at termination of contract research. Requests to access the datasets should be directed to www.dialamr.com.

## Supplementary Materials

The following supporting information can be downloaded at: https://www.mdpi.com/article/10.3390/antibiotics12050804/s1, Figure S1: Draft mastitis diagnostic guide developed by authors following Event 1, to guide veterinarians within the participating UK veterinary corporate.
