# Supplementary material for: ‘I Believe What I’m Saying More Than the Test’: The Complicated Place of Rapid, Point-of-Care Tests in Veterinary Diagnostic Practice
Source: Antibiotics (Basel). 2023 Apr 24;12(5):804. doi: 10.3390/antibiotics12050804 (PMC10215651; doi:10.3390/antibiotics12050804)

Supplementary Materials Figure S1

Draft mastitis diagnostic guide developed by authors following Event 1, to guide veterinarians within the participating UK veterinary corporate

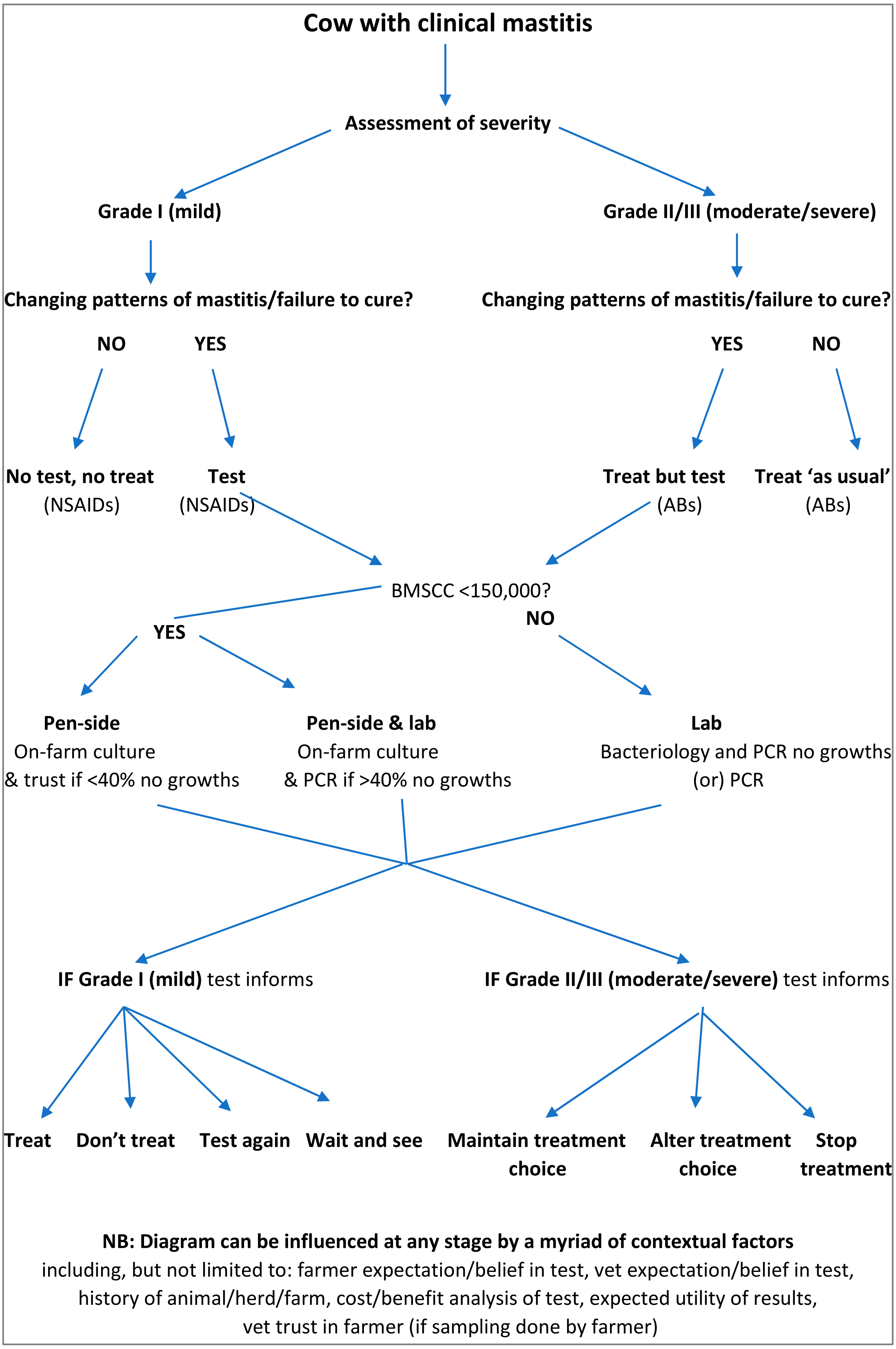

Supplement: Supplementary file 1 [file antibiotics-12-00804-s001.zip › antibiotics-2213589-supplementary.pdf]
